# Characterization and ontogeny of a novel lymphoid follicle inducer cell during development of the bursa of Fabricius

**DOI:** 10.3389/fimmu.2024.1449117

**Published:** 2024-10-21

**Authors:** Emőke Szőcs, Adam Balic, Ádám Soós, Viktória Halasy, Nándor Nagy

**Affiliations:** ^1^ Department of Anatomy, Histology and Embryology, Faculty of Medicine, Semmelweis University, Budapest, Hungary; ^2^ The Roslin Institute and Royal (Dick) School of Veterinary Studies, The University of Edinburgh, Midlothian, United Kingdom; ^3^ Department of Biochemistry and Pharmacology, Bio21 Molecular Science and Biotechnology Institute, The University of Melbourne, Parkville, VIC, Australia

**Keywords:** bursa of Fabricius, LTi cells, B cell, dendritic cell, chicken

## Abstract

The avian bursa of Fabricius (BF) is a primary lymphoid organ, where B-cell development occurs within bursal follicles of epithelial origin. During embryogenesis the epithelial anlage of the BF emerges as a diverticulum of the cloaca surrounded by undifferentiated tail bud mesenchyme. While it is believed that the epithelial-mesenchymal BF primordium provides a selective microenvironment for developing B cells, the initial events inducing lymphoid follicle formation are not fully elucidated. Using wild type and *CSF1R-*eGFP transgenic chick embryos, we find that separate B cell, macrophage and dendritic cell precursors enter the BF mesenchyme, migrate to the surface epithelium, and colonize the lymphoid follicle buds. Detailed immunocytochemical characterization revealed a novel EIV-E12+ blood-borne cell type, colonizing the surface epithelium of the BF rudiment before the entry of myeloid and lymphoid lineages and the appearance of this cell type coincides with the onset of follicle bud formation. Chick-duck chimeras and chick-quail tissue recombination experiments suggest that EIV-E12+ cells represent a transient lymphoid inducer cell population. They are not dendritic or B cells precursors, and they are capable of follicle bud induction in both dendritic cell- and B cell-depleted bursae.

## Introduction

Primary lympho-myeloid organs in endothermic Amniotes include the fetal liver, thymus, bone marrow and spleen. In avians and mammals, bone marrow is the site of embryonic and postnatal hematopoiesis (1–4). As in mammals, the avian thymus is a primary lymphoid organ where T cell maturation occurs (5, 6). However, distinct from mammals, birds possess a peculiar cloaca-associated primary lymphoid organ, the bursa of Fabricius (BF), which is required for the proliferation and differentiation of B cells (7, 8). Like other lymphopoietic tissues, the BF consists of lymphocytes, macrophages, dendritic cells and supporting stromal cells including reticular cells of mesenchymal or epithelial origin. The organ arises from ectodermal epithelium in close association with the tail bud mesenchyme and contains about 12.000 lymphoid follicles, the functional units of the BF, where generation of antibody diversity exclusively occurs (9–11). After hatching, mature B cells emigrate to the peripheral lymphoid organs and differentiate to antibody-secreting plasma cells (12–14). Similar to the avian BF, secondary lymphoid organs, like ileal Peyer’s patches in ruminants (15), the appendix of rabbits (16, 17) or the bone marrow of rodents and human (18) also support diversification of the B cell antibody repertoire, which suggests that these tissues function as mammalian bursa-equivalent organs during early postnatal development (19).

Secondary lymphoid organ development in mammals is orchestrated by lymphoid tissue inducer cells (LTi), which initiate lymph node and Peyer’s patch formation in developmentally predefined regions throughout the fetus. The tissue interactions and signaling between the LTi cells with specialized mesenchymal lymphoid tissue organizer cells (LTo) and lymphatic endothelial cells (20–22) results in the recruitment of hematopoietic cells to the developing lymphoid tissue rudiment, a mechanism that has not been characterized in primary lymphoid organs. It has recently been shown that the first blood-borne progenitors to seed the mouse thymus primordium are bipotent cells that generate an invariant T cell lineage and LTi cells, and both of these induce the maturation of thymic epithelial cells (23).

Unlike the thymus, which is colonized by three consecutive waves of T cell precursors (24), the epithelial-mesenchymal primordium of BF is colonized by blood-borne prebursal stem cells during a single receptive period from embryonic day (E) 8 to E15 (25–28). First incoming cells seed the bursa mesenchyme at E8 and express the common leukocyte antigen, CD45 (28, 29). Among these CD45+ hematopoietic cells, the first distinct cell type belongs to the Grl1+/Grl2+ granulocyte lineage that reside in the proximal part of the bursa mesenchyme, never colonize the epithelium and are no longer found after hatching (30, 31). The next CD45+ hematopoietic cells that enter the BF mesenchyme appear at E9, have stellate morphology and express colony-stimulating factor 1 receptor (CSF1R) and MHC class II antigens (32). After 72 hours, these cells migrate under the surface epithelium, develop cytoplasmic granules characteristic of bursal secretory dendritic cells (BSDC), and enter the epithelium through the basement membrane to contribute to the formation of the lymphoid follicles (27, 33). At E12, the BSDC precursors clustered in the surface epithelium start expressing CD11d and dendritic cell specific 74.3 antigen (33, 34). Invasion of the CSF1R+/74.3+ dendritic cell precursors to the surface epithelium is considered the initial step of lymphoid follicle formation, and this specialized dendro-epithelial tissue is a prerequisite of B cell colonization of the epithelial buds (35). A basophilic “dark cell” population (“dark” because they are heavily stained with toluidine blue) with polygonal morphology entering the surface epithelium of the bursal fold was previously proposed as the lymphoid follicle inducer cell (27), but the origin and fate of these cells in later developmental stages is unclear. While some studies of BF development suggest that dark cells are of mesenchymal origin, express EIV-E12 antigen and differentiate to dendritic cells (27, 36), others conclude that these cells differentiate to follicle associated epithelium (37) or contribute to the macrophage lineage (25, 38). The last phase of BF colonization occurs when chB6+/CXCR4+ B cell precursors that have already undergone somatic immunoglobulin gene rearrangement in extra-bursal sites and express sCD15 antigen, colonize the bursal mesenchyme starting at E10 and enter the developing lymphoid follicles by E13 (39, 40). These B cell precursors are responsive to the CXCL12 chemokine produced in the bursal mesenchyme, which upon emergence of the dendro-epithelial tissue shifts to the forming follicles and guides migration of the B cell precursors inside the epithelial buds (31, 41). Interestingly, in immunodeficient chicken model systems generated by CRISPR/Cas9-mediated RAG1 knockout (42) or by *in vivo* blockade of CXCR4 signaling (31), the absence of B cells did not affect follicle bud formation, suggesting the presence of lymphoid bud inducer cells.

The current study was undertaken to characterize the migration and immunophenotype of the hematopoietic cells as they colonize the BF primordia and establish the lymphoepithelial tissue in developing chicken BF. We show that, similar to mammalian LTi cells, the EIV-E12+ cells represent a distinct cell type that colonizes the bursal surface epithelium before dendritic and B cell precursors and initiates bursal lymphoid follicle formation. Results obtained from chick-duck and chick-quail chimeric experiments demonstrate that the CD45+/EIV-E12+ cells represent a transient cell population that does not differentiate to dendritic cells, macrophages, or B cells, and suggest their inducer role in bursa lymphoid follicle formation.

Testosterone-propionate induced chemical bursectomy or AMD3100 mediated inhibition of B cell immigration both show that EIV-E12+ cells, acting independently of dendritic cell and B cell precursors, have an inducer role during lymphoid follicle development. Taken together, the results of this study establish the normal pattern of migration and differentiation of hematopoietic cells in the avian BF and identify a novel lymphoid follicle inducer cell type.

## Materials and methods

### Embryos

Fertilized White Leghorn chicken (*Gallus gallus domesticus*) eggs were obtained from commercial breeders (Prophyl-BIOVO Ltd., Hungary). Transgenic *CSF1R-*eGFP-expressing chicken eggs were obtained from the National Avian Research Facility at The Roslin Institute, University of Edinburgh. Production of the *CSF1R-*eGFP reporter transgenic line has been previously described (43). Eggs were maintained at 37.5°C in a humidified incubator and embryos were staged according to the number of embryonic (E) days. After developmental staging bursa of Fabricius (BF) from E9-E13 chicken were dissected, fixed overnight in 4% buffered paraformaldehyde, and processed for immunohistochemistry. For epithelial-mesenchymal recombination experiments quail *(Coturnix japonica domestica*) eggs were purchased from commercial breeders. For chorioallantoic membrane transplantation fertilized duck eggs (*Anas platyrhynchos domesticus*) were obtained from the National Centre for Biodiversity and Gene Conservation, Institute for Farm Animal Gene Conservation, Gödöllő, Hungary. All animal experiments were approved by the Institutional Animal Care and Use Committee of Semmelweis University, Budapest, Hungary.

### Histological procedures

Tissue samples were fixed in 4% paraformaldehyde (PFA) for 1 hour at room temperature, incubated in 15% sucrose overnight at 4°C, then infiltrated with phosphate buffered saline (PBS) containing 7.5% gelatin and 15% sucrose for 2 hours at 37°C. The impregnated tissue samples were embedded in the same 7.5% gelatin medium then rapidly frozen at −50°C in 2-methylbutane (Sigma, 78-78-4). 12 μm cryosections were collected on poly-L-lysine (Sigma, P8920) coated slides and incubated with primary antibodies (**Table 1**) for 1 hour at room temperature, followed by biotinylated goat anti-mouse IgG (Vector Laboratories, BA-9200) secondary antibody and avidin-biotinylated peroxidase complex (ABC; Vectastain Elite ABC kit, Vector Laboratories, PK-6100). Endogenous peroxidase activity was quenched by 3% hydrogen-peroxide (Sigma, H1009) in PBS for 10 minutes. The binding sites of the primary antibodies were visualized by 4-chloro-1-naphthol (Sigma, C8890).

**Table 1:** List of primary antibodies.

Antigen	Clone	Structure/cell identified	Dilution	Source of antibody	Isotype
CD45	HISC7	hematopoietic cells	1:200	Prionics Co.	mouse IgG_2A_
CD4	CT-4	T cells	1:200	Southern Biotech	mouse IgG_1_
200 kDa glycoprotein	EIV-E12	lymphoid follicle inducer cells	supernatant	Dr. Todd Pharr (USA) (36)	mouse IgG_1_
chB6 (Bu-1a/b)	BoA1	B cells	supernatant	Southern Biotechnology Associates, Birmingham, USA	mouse IgG_1_
IgM	M2	B cells	1:200	Dr. Sonja Hartle, München University (44)	mouse IgG_1_
CXCR4	9D9	B cells, granulocytes	supernatant	Dr. Sonja Hartle, München University	mouse IgG_2A_
CSF1R	ROS-AV-170	dendritic cells, macrophages	1:100	Dr. Zhiguang Wu, Roslin Institute (45)	mouse IgG_1_
TIM4	JH9	macrophage subsets	1:1000	Dr. Zhiguang Wu, Roslin Institute (46)	mouse IgG_1_
74.3	CVI-ChNL-74.3	chicken dendritic cells	1:50	Prionics	mouse IgG_1_
MHCII	21-1A6	chicken major histocompatibility complex class II expressing antigen presenting cells	1:100	Thermo Fisher	mouse IgG_1_
duck CD8	CD8-1	duck leukocytes	1:200	Dr. Sonja Hartle, München University (47)	mouse IgG_1_
E-cadherin	36	epithelial cells	1:200	BD Biosciences	mouse IgG_2A_
cytokeratin	Lu-5	epithelial cells	1:200	Sigma	mouse IgG_1_
Neuropilin-1	TB2	endothelial cells	supernatant	DSHB	mouse IgG_1_
motor neuronal marker (SC-1)	BEN	bursa epithelium and neurons	supernatant	DSHB	mouse IgG_1_
quail cell nuclear antigen	QCPN	all quail cells	supernatant	DSHB	mouse IgG_1_

For double immunofluorescence, sections were incubated with primary antibodies (**Table 1**) for 1 hour at room temperature followed by Alexa-conjugated fluorescent secondary antibodies: Alexa Fluor 594 goat anti-mouse IgG (1:200, A32742), Alexa Fluor 488 goat anti-mouse IgG (1:200, A32723), Alexa Fluor 488 goat anti-mouse IgG2a (1:200, A21131), Alexa Fluor 594 goat anti-mouse IgG2a (1:200, A21135), Alexa Fluor 488 goat anti-rabbit IgG (1:200, A32731), all from ThermoFisher Scientific. Cell nuclei were stained with 4,6-diamidino-2-phenylindole (DAPI; Vector Labs, Burlingame, California) and sections were covered by aqueous Poly/Mount (Polyscience Inc. Warrington, PA, 18606).

Section images were recorded using a Nikon Eclipse E800 fluorescence microscope and Zeiss LSM 710 confocal microscope. Image processing was performed using CellSens, ZEISS ZEN Imaging proprietary software and ImageJ. Images were compiled using Adobe Photoshop 7.0.

### Immunoelectron microscopy

E10 bursa of Fabricius were fixed and embedded in 7.5% gelatine as described above. Gelatine cubes were postfixed in 4% paraformaldehyde for 48 hours at 4°C. 200 μm thick vibratome sections were collected and incubated overnight with EIV-E12 primary antibody, followed by biotinylated goat anti-mouse IgG secondary antibody overnight at 4°C and avidin-biotinylated peroxidase complex for 1 hour at room temperature (Vectastain Elite ABC kit, Vector Laboratories, PK-6100). Endogenous peroxidase activity was quenched with 3% hydrogen peroxide (Sigma, H1009). EIV-E12 staining was visualized with 3,3′-diaminobenzidine-NiSO_4_ (Sigma). Samples were postfixed in 4% glutaraldehyde for 24 h and 1% osmium tetroxide (Polysciences Inc., Warrington, PA) for 2 h. After dehydration in graded ethanol, tissue blocks were embedded in Polybed/Araldite 6500 (Polysciences Inc., Warrington, PA). Semithin (1 μm) sections were stained with toluidine-blue; ultrathin (60 nm) sections were contrasted with uranyl acetate and lead citrate and studied with a JEOL electron microscope type JEM-1200EX.

### Chorioallantoic membrane transplantation

Chorioallantoic membrane (CAM) grafts were generated as previously described (32). To follow the development of EIV-E12+ cells in the bursal follicles, E9 chicken bursa of Fabricius were isolated in 5% Penicillin/Streptomycin (Sigma, P0781) containing PBS, transplanted onto the CAM of E11 duck embryos and further cultured for 7 and 11 days (n=8). For CXCR4 signaling blocking experiments 1 μl of 200 μM AMD3100 (Sigma, A5602) was injected into the mesenchymal wall of isolated E9 bursa primordia (31). Grafts were cultured on the CAM of E9 chicken embryos for 9 days (n=9). Control bursa CAM grafts were generated by PBS injection, used as solvent in the experimental samples (n=6). CAM grafts were excised, fixed in 4% paraformaldehyde and embedded in gelatin/sucrose for cryosectioning.

### Chemical bursectomy with testosterone-propionate treatment

Testosterone-propionate treatment was carried out as previously described (27). Briefly, fertilized White Leghorn eggs were incubated for 24 hours at 37.5°C, eggs were dipped in 2,5% testosterone propionate (TP, Sigma, 57-85-2) dissolved in absolute ethanol (Molar Chemicals Kft., 02910-101-340) for 5 seconds (**Figure 6A**). Treated eggs were returned to the incubator and further incubated up to 14 days. BF from control and TP-treated embryos were isolated (n=8), fixed in 4% PFA, embedded in gelatin/sucrose and processed for cryosectioning.

### Epithelial-mesenchymal tissue recombination experiments

To assess the follicle bud inducing capacity of EIV-E12+ cells, chick-quail epithelial-mesenchymal tissue recombination experiments were performed as described before (11). Precolonized E8 BF epithelium was recombined with mesenchyme of BF or hindgut origin. Briefly, E8 quail BF primordium, E9 chicken BF primordium and E9 chicken hindgut were isolated and digested with 0.03% collagenase (Sigma, T1875) in Dulbeccos’s Modified Eagles Medium (DMEM, Sigma, D6429) for 15 minutes at 37°C. Enzymatic activity was blocked by three washing steps in fetal bovine serum (FBS, Sigma, F9665). The BF epithelium was isolated from the mesenchyme using fine forceps. In case of hindgut, the mesenchymal wall of the tissue was longitudinally opened and the intestinal epithelium was removed with the help of a tungsten needle. To allow the tissues to adhere tissue recombinants (Experiment 1: quail BF epithelium+chicken BF mesenchyme; Experiment 2: quail BF epithelium+chicken hindgut mesenchyme) were cultured on a layer of 3D-collagen gel matrix (Gibco, A10483). After overnight incubation, the recombinant chimeric tissues were removed from the collagen gel and transplanted onto the CAM of E9 chicken then cultured for 9 days. CAM grafts were excised, fixed in 4% paraformaldehyde and embedded in gelatin/sucrose for cryosectioning.

### Statistical analysis

Statistical analysis was performed by Mann–Whitney *U* test, violin plots presenting all data points were generated using the Graphpad Prism v9.4.1 proprietary software. Lines represent the median of all data points. P<0.05 was considered significant.

## Results

### Colonization of the bursa of Fabricius by hematopoietic cells

The epithelial-mesenchymal primordium of the chicken BF arises at around E5 in the tail bud mesenchyme as a dorsal pouch of the cloacal epithelium (11). Colonization of the bursal epithelial anlage by hematopoietic cells is a prerequisite for the formation of the lymphoid follicles where B cell differentiation occurs. Although the main blood-borne population of the developing BF is represented by the B cells, previous experiments have demonstrated that seeding of multiple myeloid cells also takes place in the bursa primordium that precedes the entry of B cell precursors (11, 29, 48). In order to characterize the immunophenotype of hematopoietic cells colonizing the bursa primordium, comparative immunostainings were performed using a panel of commercially available lymphocyte-, and myeloid-specific markers: anti-CD45 monoclonal antibody (mAb), also known as common leukocyte antigen, specific for all nucleated cells of hematopoietic origin (32, 49); Grl1/2, present in myeloid progenitors and granulocytes (50); MHC II, expressed by antigen-presenting cells and their precursors (51, 52); CSF1R (colony stimulating factor-1 receptor), also known as CD115, present on the surface of dendritic cells, macrophages and bursal follicle associated epithelium (52–54); anti-74.3 mAb, a chicken dendritic cell marker (28, 55); anti-phosphatidylserine receptor TIM4, specific for macrophages (46); anti-EIV-E12, an antigen expressed by 90% of adult bursal cells (36); chB6, pan-B cell marker (56); anti-BAFF-receptor (BAFFR), also known as CD268, a cell surface antigen expressed by mammalian and avian B cells (57), and anti-CD4, and -CD8 antibodies, recognizing the chicken T cells (58).

**Figure 1:**
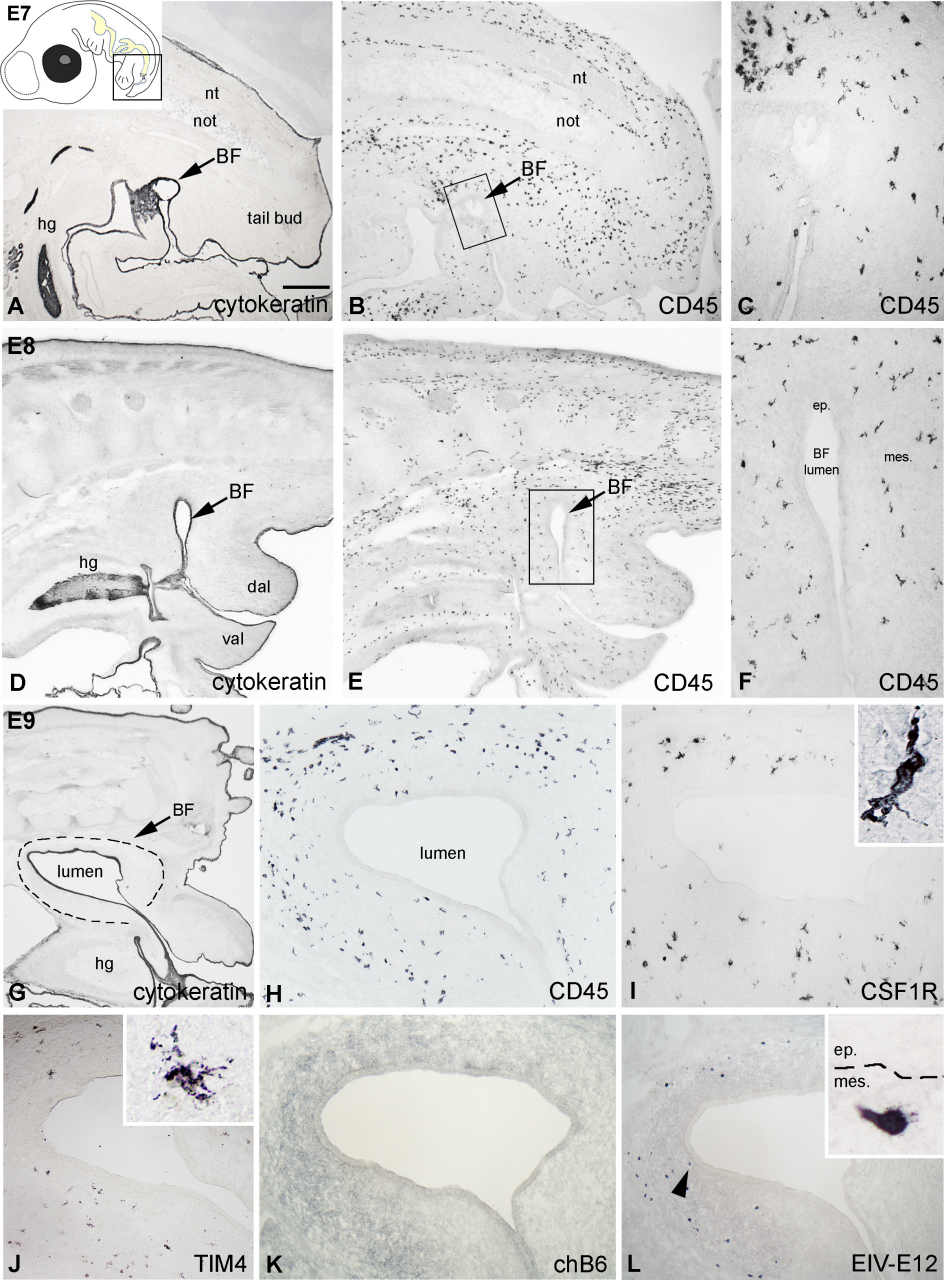
Hematopoietic colonization of the chick embryonic bursa. **(A)** The cytokeratin+ epithelial anlage of the BF appears on E7 and remains in contact with the proctodeum (arrow). **(B, C)** At this developmental stage CD45+ hematopoietic cells uniformly colonize the tail bud mesenchyme. Outlined area in B is magnified in C. **(D-F)** On E8 CD45+ cells are scattered in the bursal mesenchyme. Outlined area in E is shown in **F**. **(G)** On E9 the bursal lumen is lined by the cytokeratin+ surface epithelium. **(H)** CD45+ hematopoietic cells possessing round and ramified morphology are scattered throughout the bursal mesenchyme. Outlined area marks the BF. **(I, J)** Two distinct populations of cells with ramified morphology occur in the external part of the mesenchyme: CSF1R+ cells are more numerous than the TIM4+ cells. **(K)** chB6+ B cell precursors are not present in the bursa at this stage. **(L)** The EIV-E12 mAb labels round cells in the E9 bursa, some of which are in close proximity to the surface epithelium (arrowhead). nt, neural tube; not, notochord; hg, hindgut; BF, bursa of Fabricius; dal, dorsal anal lip; val, ventral anal lip; ep, epithelium; mes, mesenchyme. Arrows indicate the bursal primordium. Scale bar: 400 µm **(A, B)**, 150 µm **(C)**, 400 µm **(D, E)**, 170 µm **(F)**, 300 µm **(G)**, 120 µm **(H-L)**, 20 µm [**(I, J)** insets], 12 µm [**(L)** inset].

At E7, the epithelial rudiment of the chicken bursa starts growing into the tail bud mesenchyme (**Figure 1A**), which at this stage is already colonized by CD45+ hematopoietic cells (**Figures 1B, C**). Twenty-four hours later, the bursal anlage develops a luminal compartment lined by the cytokeratin+ epithelium (**Figure 1D**) and the number of CD45+ cells showing both round and ramified morphology increases steadily in the bursa mesenchyme (**Figures 1E, F**). On E9, CD45+ cells appeared more numerous, and some cells were localized near the surface epithelium (**Figures 1G, H**). Immunostaining performed on consecutive sections revealed that ramified cells expressing CSF1R and an amoeboid cell population immunoreactive for TIM4 occur in the external mesenchyme of the BF (**Figures 1I, J**). While hematopoietic cells seed the bursa in large numbers by E9, B cells expressing the chB6 antigen are absent at this early stage (**Figure 1K**). In contrast, immunostaining performed with the EIV-E12 mAb, which recognizes a 200 kDa membrane glycoprotein on the surface of adult lymphoid cells, labeled a round cell type with a single spike-like process, scattered in the bursal mesenchyme (**Figure 1L**). On the other hand, cells expressing other B and T lymphocyte-specific markers such, BAFFR, CD4, or CD8 were not detectable (data not shown).

### EIV-E12+ cells colonize the bursa epithelium and induce follicle bud formation

**Figure 2:**
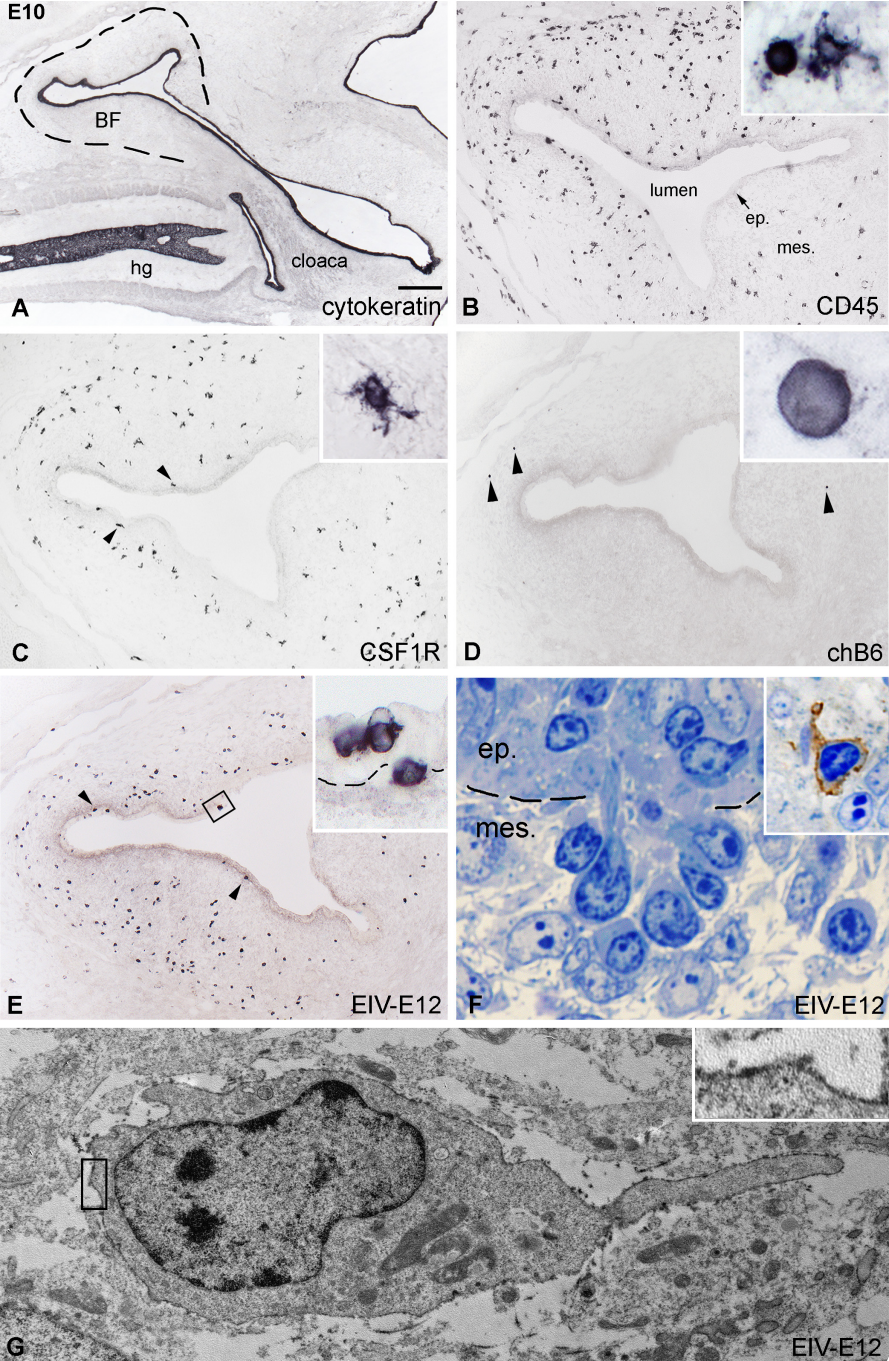
Initiation of follicle bud formation. **(A)** The cytokeratin+ epithelial anlage of the E10 BF is a vesicle-like structure (dashed line) surrounded by the tail bud mesenchyme. **(B)** CD45+ hematopoietic cells (inset) are present in large numbers in the bursal mesenchyme and some of the cells reach the surface epithelium. **(C)** CSF1R+ ramified cells are localized in the bursal mesenchyme, a few cells present under the surface epithelium (arrowheads). **(D)** Presence of chB6+ B cell precursors in the bursal rudiment can be first observed on E10 in the peripheral mesenchyme (arrowheads). **(E)** Many EIV-E12+ cells are grouped under the surface epithelium (arrowheads), cross the basement membrane and induce the formation of follicle buds. Outlined area is magnified in inset. **(F)** Toluidine blue stained 1 μm thin section of a group of darkly stained cells crossing the basement membrane of the surface epithelium. Dashed line marks the BM. Inset: EIV-E12 immunohistochemistry on semithin section. **(G)** Immunoelectron microscopy of EIV-E12+ cell shows a poorly developed cytoplasm with few organelles, euchromatic nucleus and a single cellular process pointing towards the surface epithelium. Brown chromogen outlined in inset marks the EIV-E12 immunoreactivity. ep, epithelium; mes, mesenchyme; hg, hindgut; BF, bursa of Fabricius. Scale bar: 300 µm **(A)**, 120 µm **(B-E)**, 10 µm [**(B)** inset], 14 µm [**(C)** inset], 5 µm [**(D)** inset], 8 µm [**(E)** inset], 4 µm **(F)**, 5 µm (**F** inset), 800 nm **(G)**, 180 nm [**(G)** inset].

By E10, the CD45+ hematopoietic cells colonize the surface epithelium (**Figures 2A, B**). According to histological studies in developing chicken, quail and guinea fowl BF, blood-borne basophilic “dark” cells appear in the mesenchyme, aggregate under the epithelium, and migrate through the basement membrane, which is followed by follicle bud formation (27, 29, 59, 60). The epithelial bud represents the primordium of the medulla of the bursal lymphoid follicles, and basophilic dark cells are proposed to play a role in follicle formation by preparing the epithelium for seeding by the incoming B cell precursors. To determine the immunophenotype of dark cells, we stained consecutive E10 BF sections using anti-CD45 and lympho-myeloid cell-specific antibodies. At this developmental stage, CD45+ cells showed round morphology, accumulating under the basement membrane and entering the surface epithelium (**Figures 2A, B**). Previous reports indicated that the first cells migrating into the bursa are granulocytes, macrophages, and dendritic cell precursors (28, 29, 48). Therefore, follicle buds were further analyzed for the presence of myeloid cells expressing either bursa secretory dendritic cell-specific 74.3 antigen, the CSF1R receptor characteristic for macrophages and dendritic cells, and the macrophage-specific TIM4 molecule. During this developmental stage only few CSF1R+ and TIM4+ myeloid cells were present in the bursa mesenchyme, uniformly scattered throughout the developing folds, but colonization of the surface epithelium cannot be observed (**Figure 2C**). Occasional chB6+ B cells may be seen in the external bursal mesenchyme (**Figure 2D**). On parallel sections, EIV-E12 mAb recognized the same group of CD45+ intraepithelial cells (**Figure 2E**; **Supplementary Figure S1A-A”**), suggesting that epithelial cell clusters detected by EIV-E12 mAb correspond to previously described basophilic dark cells (**Figures 2E, F**). Immunocytochemistry on semithin sections and EIV-E12 immunoelectron microscopy indeed demonstrate the dark cell specificity of the EIV-E12 antigen and confirm the cell-cell contact between dark cells and the surface epithelium (**Figures 2F, G**). EIV-E12 immunoreactive cells characterized by a spikelike process were detected by electron microscopy using diaminobenzidine, which appears as a black precipitate on the cell surface (**Figure 2G**, inset).

### Colonization of the follicle buds by dendritic cells, macrophages and B cells

**Figure 3:**
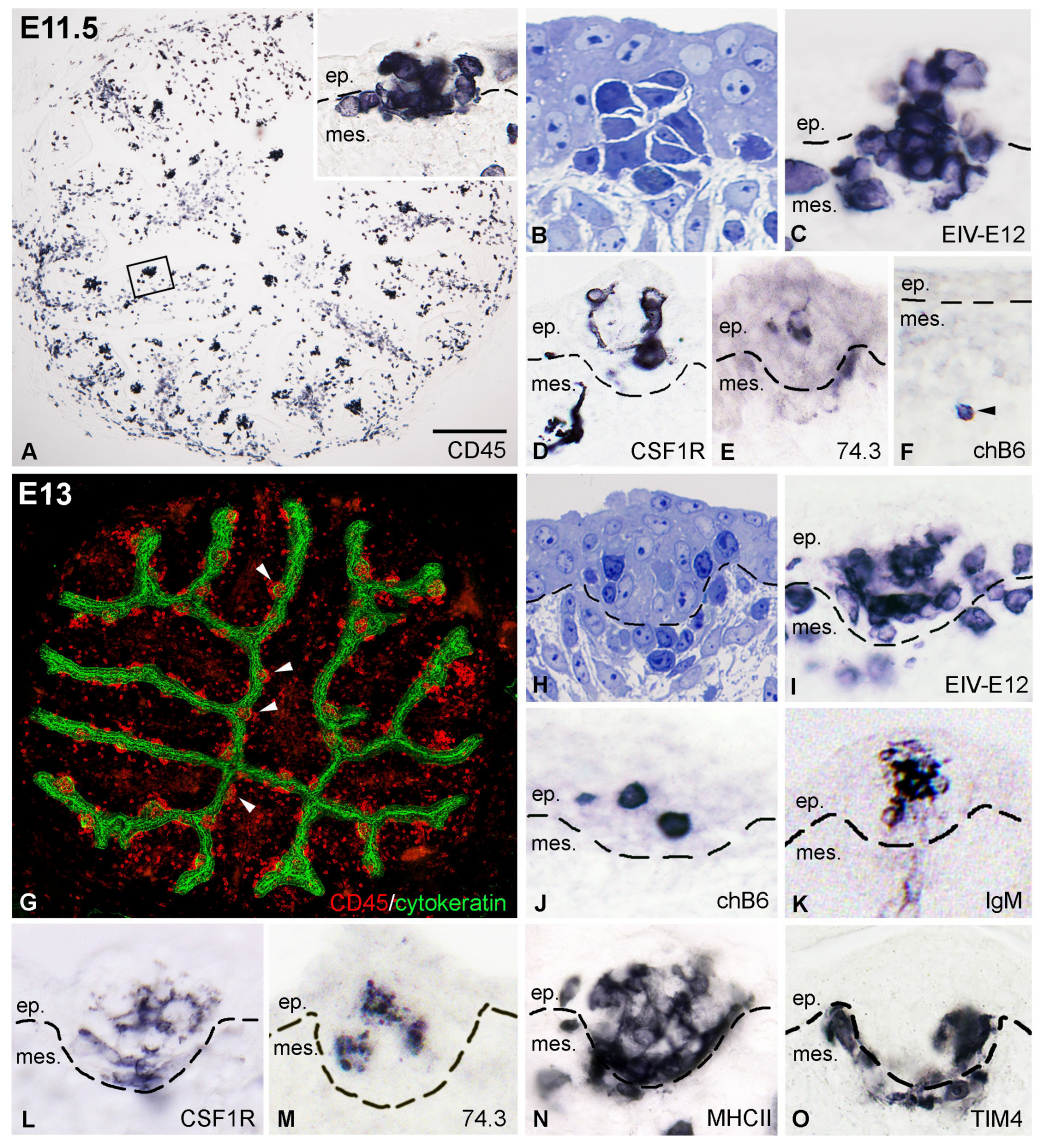
Colonization of the follicle buds by dendritic cells, macrophages and B cell precursors. **(A)** At E11.5 CD45+ cells are present in large numbers in the axis and the surface epithelium of the bursal folds. The outlined area is shown in inset. **(B)** Toluidine blue stained semithin section of a group of basophilic cells entering the surface epithelium. **(C)** Developing follicle buds are filled with round EIV-E12+ cells. **(D)** CSF1R+ dendritic cell precursors first colonize the developing follicle buds on E11.5. **(E)** Ramified cells reaching the surface epithelium express the 74.3 bursal secretory dendritic cell specific antigen. **(F)** At this stage only few chB6+ B cell precursors (arrowhead) are present in the bursa mesenchyme, distant form the surface epithelium. **(G)** On E13 the newly formed cytokeratin+ follicle buds (arrowheads) and the mesenchymal core of the bursal folds are infiltrated with CD45+ hematopoietic cells. **(H)** Toluidine blue stained semithin section of an E13 follicle bud. **(I)** EIV-E12+ cells are grouped inside the developing follicles. **(J, K)** chB6+ B cell precursors first colonize the follicle buds on E13 and express surface IgM. **(L-O)** Some of the cells in the follicle buds express dendritic cell specific markers such as CSF1R **(L)**, 74.3 **(M)**, MHCII **(N)** and macrophage specific receptors such as the TIM4 **(O)**. Dashed lines mark the epithelial basement membrane. ep, epithelium; mes, mesenchyme. Scale bar: 100 µm **(A)**, 40 µm (A inset), 15 µm **(B-E)**, 50 µm **(F)**,150 µm **(G)**, 20 µm **(H-O)**.

By E11.5 proliferation of the mesenchyme results in the formation of bursal folds with many well-defined follicle buds containing CD45+ cell clusters (**Figure 3A**). Some of these cells were aggregated in follicle buds similar to basophilic dark cells (**Figure 3B**), while others were scattered over the bursal mesenchyme. Immunocytochemistry reveals that EIV-E12 is strongly expressed by cells colonizing the surface epithelium (**Figure 3C**). Highly ramified CSF1R+ cells were observed in the mesenchyme, some of which reach the epithelial surface and colonize the follicle buds (**Figure 3D**). chB6+ B cells were very few in number and limited to the mesenchyme, far from the surface epithelium (**Figure 3F**). E11.5 bursal sections were also stained with 74.3 chicken dendritic cell specific antibody. This demonstrated that similar to CSF1R-expressing cells, 74.3+ precursors of bursal dendritic cells cross the basement membrane and colonize the follicle buds (**Figure 3E**). Hematopoietic cell colonization is almost complete by E13. At this stage, both the cytokeratin+ surface epithelium and the bursal mesenchyme are heavily infiltrated by CD45+ cells (**Figure 3G**). EIV-E12+ cells fill the follicle buds (**Figures 3H, I**). chB6+ B cells first colonize the epithelial buds on E13 (**Figure 3J**), which coincides with the expression of membrane IgM, detectable only on the surface of B cells in the follicular microenvironment (**Figure 3K**). Immunostaining for CSF1R, 74.3, MHCII and TIM4 demonstrated that ramified cells entering the follicle buds express dendritic cell and macrophage specific markers (**Figures 3L-O**).

### Folliculogenesis in *CSF1R-*eGFP chicken embryos

**Figure 4:**
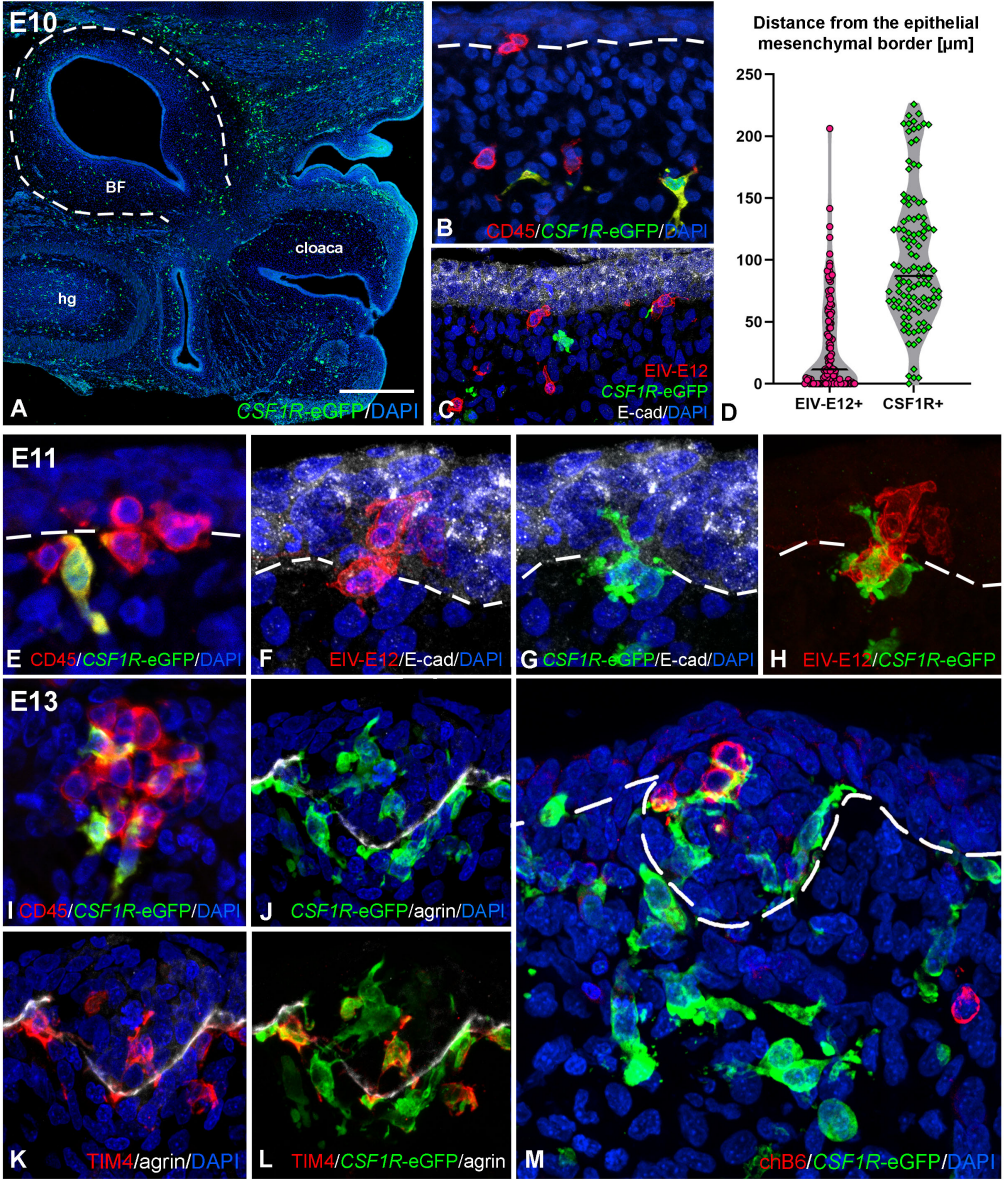
Folliculogenesis in *CSF1R-*eGFP transgenic chicken embryos. **(A)** Longitudinal sections of E10 embryo tail bud shows *CSF1R-*eGFP+ cells scattered in the BF mesenchyme. **(B)** Double staining with CD45 specific antibody revealed that CD45+/*CSF1R-*eGFP- cells of round morphology reach the bursal surface epithelium, whereas CD45+/*CSF1R-*eGFP+ cells of ramified morphology are restricted to the bursal mesenchyme. **(C)** EIV-E12+ cells colonize the E-cadherin+ surface epithelium. EIV-E12+ cells do not express *CSF1R-*eGFP. **(D)** Quantification of the distance (µm) of colonizing EIV-E12+ and CSF1R-eGFP+ cells from the surface epithelium. Lines represent the median of the values. **(E–H)** Consecutive sections of E11 *CSF1R-*eGFP BF shows the ramified cells colonizing the surface epithelium. Epithelial colonization by EIV-E12+ cells is followed by the entry of *CSF1R-*eGFP expressing cells. **(I)** At E13 the follicle buds contain CD45+ cells of both round and ramified morphology. **(J-L)** Two distinct ramified cell populations can be distinguished inside the forming follicles: *CSF1R-*eGFP+/TIM4- dendritic cell precursors and *CSF1R-*eGFP+/TIM4+ macrophage precursors. **(M)** chB6+ B cells first colonize the follicle buds on E13. Outlined area in A marks the BF. Dashed lines mark the epithelial basement membrane in **(B, E-H, M)**. hg, hindgut; BF, bursa of Fabricius. Scale bar: 340 µm **(A)**, 30 µm **(B, C)** 15 µm **(E-H, M)**, 20 µm **(I-L)**.

To characterize the earliest stages of follicle bud formation, we followed the hematopoietic colonization of the bursal surface epithelium using *CSF1R-*eGFP transgenic chicken embryos. The *CSF1R-*eGFP reporter transgenic chicken strain was used to visualize macrophages and dendritic cells (43, 45). On E10, at the onset of bursal hematopoietic colonization, *CSF1R-*eGFP-expressing ramified cells are uniformly scattered in the mesenchyme of the tail bud and the developing bursal anlage (**Figure 4A**). Double immunostaining for CD45, EIV-E12 and GFP shows the presence of isolated CD45+/*CSF1R-*eGFP+ cells in the bursa mesenchyme, distant from the surface epithelium, while a population of CD45+/*CSF1R-*eGFP- cells reach the surface epithelium on E10 (**Figure 4B**). Cells that belong to the CD45+/*CSF1R-*eGFP- population uniformly express the EIV-E12 antigen and colonize the E-cadherin+ surface epithelium (**Figure 4C**). The number and distance of colonizing cells to the surface epithelium was quantified in 30 fields of view, with an area of 250 μm^2^ perpendicular to the surface epithelium. We measured the distance of immunolabeled EIV-E12+ and *CSF1R-*eGFP cells from the epithelial mesenchymal border starting from the nucleus of each cell. On E10, EIV-E12+ cells are at an average distance of 30.59 μm, while *CSF1R-*eGFP cells at 101.29 μm from the surface epithelium (**Figure 4D**). On E11, well-defined CD45+ cell clusters are present at the level of the surface epithelium, among which CD45+/*CSF1R-*eGFP+ ramified cells begin to appear (**Figure 4E**). *CSF1R-*eGFP expressing cells infiltrate the bursal epithelium in a sequential manner, specifically in those areas where EIV-E12+/*CSF1R-*eGFP- cells entered the epithelium (**Figures 4F-H**). Double-immunofluorescence labeling demonstrated that EIV-E12+/CD45+/*CSF1R-*eGFP- cells with round morphology and EIV-E12-/CD45+/*CSF1R-*eGFP+ ramified cells shape the follicular microenvironment (**Figure 4I**). To characterize the phenotype of the ramified myeloid cell populations in the epithelial buds, immunostaining for TIM4 was combined with endogenous GFP detection. Double immunofluorescent staining shows that a subpopulation of *CSF1R-*eGFP positive cells co-express the macrophage-specific TIM4 antigen, while a distinct cell population is *CSF1R-*eGFP+/TIM4- (**Figures 4J–L**). Immunostaining specific for agrin, a heparan sulfate proteoglycan present in basement membranes (61), lines the developing follicle buds, separating the epithelial and mesenchymal compartments, which in adult birds correspond to the medullary and cortical regions of lymphoid follicles (11, 28). Both types of myeloid cells are uniformly localized inside and outside the agrin outlined developing follicles (**Figures 4J-L**). In accordance with previous observations (**Figure 3J**), chB6+ B cells first colonize the follicle buds starting from E13, with several B cell precursors present at the level of the epithelium intermingled with *CSF1R-*eGFP+ ramified cells (**Figure 4M**).

### Duck-chicken chimera reveals that EIV-E12+ cells are not precursors for dendritic or B cells

Based on our immunocytochemical observations, invasion of the EIV-E12+ cells into the surface epithelium of the embryonic BF coincides with the induction of the epithelial follicle buds. Considering that EIV-E12+ cells appear first in the bursa mesenchyme at E9, together with CSF1R+ and TIM4+ myeloid cells, the question arises whether EIV-E12+ cells represent a precursor cell for the CSF1R+ dendritic cell lineage, a hypothesis which has been raised several times by previous publications (33, 36). In addition, in later developmental stages after lymphoid colonization of the follicle buds is completed, B cells also express the EIV-E12 antigen, which raises the possibility that EIV-E12+ cells may represent the precursor for chB6+ B cells (36, 56, 62).

**Figure 5:**
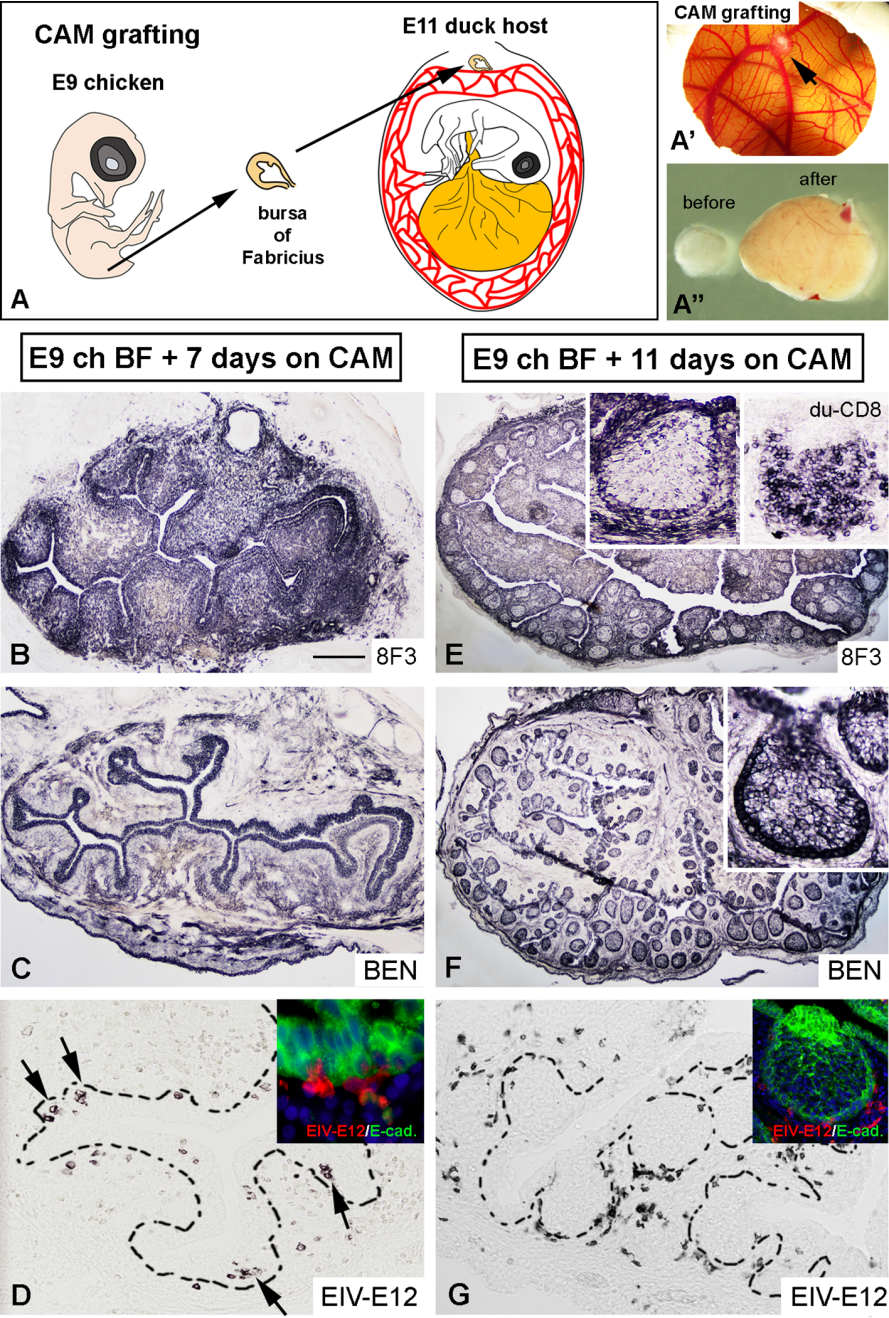
Duck-chicken chorioallantoic membrane transplantation reveals that EIV-E12+ cells are not precursors for dendritic cells or B cells. **(A)** To follow the fate of EIV-E12+ cells in the developing bursal follicles, E9 chicken bursa of Fabricius were cultured for up to 11 days on E11 duck CAM (n=8). **(A’)** Chicken bursa graft on the CAM after 11 days of incubation (arrow). **(A”)** Comparison of bursal size before and after chorioallantoic membrane transplantation. **(B)** 8F3 chicken cell specific mAb marks the developing epithelial-mesenchymal rudiment of the bursa. **(C)** Bursal epithelium shows BEN immunoreactivity. **(D)** Consecutive section shows EIV-E12 immunoreactive cells colonizing the surface epithelium (arrows). Example of EIV-E12+ cells entering the E-cadherin+ surface epithelium shown in inset. **(E, F)** 11 days after CAM transplantation the BF develops several folds, filled with lymphoid follicles, which are colonized by CD8+ leukocytes of duck origin (inset **Figure 4E**). **(G)** Few EIV-E12+ cells are present at the margin of the follicles and many are excluded to the interfollicular space. Scale bar: 6 mm **(A’)**, 1 mm **(A”)**, 250 µm **(B, C)**, 50 µm **(D)**, 30 µm [**(D)** inset], 350 µm **(E, F)**, 150 µm **(G)**, 70 µm [**(E–G)** inset].

To determine whether EIV-E12+ cells colonizing the BF rudiment later differentiate to either dendritic cells or B cells, E9 chicken BF were isolated and transplanted onto the chorioallantoic membrane (CAM) of E11 duck embryos (**Figures 5A, A’**). At this stage migrating EIV-E12+ cells were present in the bursa mesenchyme and have not yet colonized the epithelium. CAM grafts were cultured *in ovo* for 7 and 11 days (**Figure 5A”**), serially sectioned and analyzed for the presence of EIV-E12+ cells, CSF1R+ dendritic cells and chB6+ B cells using species specific antibodies. After 7 days of incubation, proliferation of the mesenchyme resulted in the development of bursal folds (**Figure 5B**), lined by the surface epithelium, that expresses BEN, a cell surface molecule of the immunoglobulin superfamily, specific during avian development for selective sets of neurons and for the epithelium of the bursa of Fabricius (**Figure 5C**) (63). CD45+ hematopoietic cells of chicken origin are present in large numbers in the graft, many of which are found clustered in the surface epithelium. EIV-E12 immunolabeling reveals that, similar to control embryonic bursa rudiments, EIV-E12+ cells in the graft have colonized the surface epithelium in multiple areas (**Figure 5D**). By 11 days post-transplantation, numerous follicles have differentiated. In the transplanted CAM grafts 8F3 (a chicken cell specific monoclonal antibody) immunoreactivity labels the chicken derived epithelial and mesenchymal compartments (**Figure 5E**). The 8F3+ epithelial cells express BEN (**Figure 5F**) and E-cadherin (**Figure 5G** inset) and form the reticulum of the chicken derived follicles, which are colonized by CD8+ leukocytes of duck origin (**Figure 5E** inset). It has been described that adult duck bursal B cells express the CD8 antigen (47). Immunostaining shows a distinct localization of EIV-E12+ cells, which in contrast to their clustered phenotype inside the developing follicle buds, in later embryonic stages are restricted to the interfollicular connective tissue, with a few cells present at the peripheral regions of the follicular medulla (**Figure 5G**, inset), indicating that EIV-E12 cells do not differentiate to B cells or dendritic cells. Neither chicken derived CSF1R+ nor chB6+ cells can be found in these chimeric follicles, suggesting the duck origin of these cells (data not shown).

We conclude that, EIV-E12+ cells represent a transient cell population in early bursal development, their primary role being the colonization of the surface epithelium before lymphoid follicle formation. Furthermore, the embryonic EIV-E12+ cells are not dendritic cells or B cell precursors and do not represent a definitive cell population of the bursal follicles.

### Follicle buds develop in the absence of dendritic and B cell precursors

**Figure 6:**
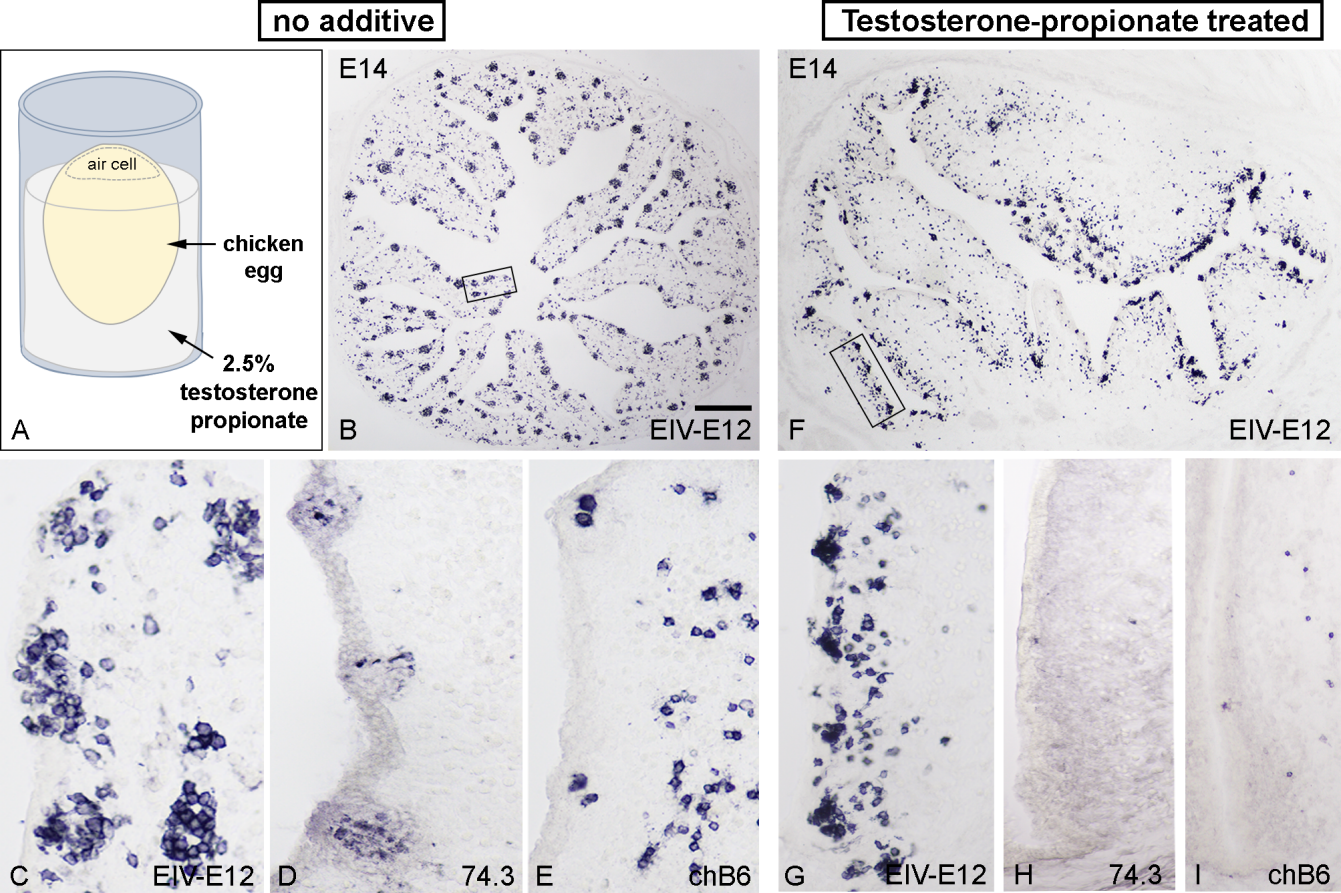
Testosterone-propionate treatment inhibits dendritic cell differentiation but does not influence follicle bud formation. **(A)** To test whether the absence of dendritic cells could affect follicle formation, one day old chicken eggs were treated with 2.5% testosterone propionate and further incubated until E14. **(B)** Control bursae show normal follicle development (n=8), with EIV-E12+ cells filling the epithelial buds along the bursal folds. Boxed area magnified in **(C)**. General view from E14 bursa showing the presence of EV-E12+ cells. **(D)** 74.3+ dendritic cells and **(E)** chB6+ B cells colonize the developing follicle buds. **(F)** Testosterone treatment does not inhibit EIV-E12+ cell colonization of the surface epithelium (n=8). Boxed area magnified in **(G)**. Compared with control bursae, EIV-E12+ follicle buds develop in large numbers along the axis of the folds. **(H)** In contrast, 74.3+ dendritic cells are absent in testosterone treated bursae. **(I)** Few chB6+ B cells are present in the mesenchyme of the folds and occasionally migrate into the surface epithelium. Scale bar: 280 µm **(B)**, 50 µm **(C-E)**, 230 µm **(F)**, 75 µm **(G-I)**.

Using chick-quail chimeric studies, we previously described that the colonization of the bursa epithelium is a two-steps process: 74.3+ dendritic cell precursors of blood-borne origin enter the surface epithelium and form a transitory dendro-epithelial tissue, which is able to receive the chB6+ B cell precursors (60). Formation of the dendro-epithelial tissue and differentiation of bursal dendritic cells are highly sensitive to testosterone propionate treatment (64), which results in bursectomy by preventing the differentiation of 74.3+ bursal dendritic cells and subsequently the normal development of the BF. Therefore, to study the role of EIV-E12+ cells in the absence of dendritic cells, testosterone mediated chemical bursectomy was performed by dipping one-day-old incubated eggs into 2.5% testosterone solution and the embryos were allowed to develop *in ovo* for 13 days (**Figure 6A**). On the 14th day of incubation, a significant number of cells express the EIV-E12 antigen, which are grouped in the follicular buds of the control and testosterone treated BF epithelium (**Figures 6B, C, F, G**). During follicular development, bursal dendritic cells differentiate and express 74.3 dendritic cell antigen (**Figure 6D**), while testosterone-treated bursae show no sign of 74.3 immunoreactive dendritic cells (**Figure 6H**). At this stage, scattered chB6+ B cells occur throughout the mesenchyme of the folds and occasionally migrate into follicles, but the histological difference remains remarkable between control and testosterone-treated embryonic bursae (**Figures 6E, I**).

We recently showed that injection of the selective CXCR4 antagonist AMD3100 into the bursa mesenchyme completely inhibited migration of chB6+ B cell precursors from the mesenchyme to the surface epithelium, while immigration of CSF1R+ dendritic cell precursors into the follicle bud was not affected (31). To test whether the absence of B cells could affect follicle formation, BF primordium dissected from E9 embryos were injected with AMD3100 or PBS (control) and transplanted on the CAM of E9 chicken embryos for additional 9 days (**Supplementary Figure S2**). Consecutive sections of control and AMD3100 treated CAM grafts were stained for chB6, EIV-E12 and 74.3 antigens. As expected, immunostaining on control CAM graft shows that chB6+ B cells fill up the follicle buds (**Supplementary Figures S2A-C**) and express the EIV-E12 antigen (**Supplementary Figure S2D**). 74.3 monoclonal antibody marks the bursal dendritic cells inside the follicle buds (**Supplementary Figure S2E**). CAM grafts grown in the presence of AMD3100 demonstrated migratory arrest of the chB6+ B cells (**Supplementary Figures S2F, H**), but immigration of EIV-E12+ cells and 74.3+ dendritic cell precursors into the follicle bud was not affected (**Supplementary Figures S2G, I, J**). Taken together, these results demonstrate that EIV-E12+ cells, independent of dendritic cell and B cell precursors, play an important role in follicle bud development within the bursa of Fabricius.

### EIV-E12+ cells induce follicle bud formation

Given the observation that EIV-E12+ cells are present in the BF mesenchyme and colonize the organ primordia before the entry of dendritic and B cell precursors, we hypothesized that EIV-E12+ cells are the tissue inducer cells (LTi cells) for lymphoid follicle formation. In humans and mice the LTi cells of developing lymph nodes and Peyer’s patches are a heterogenous population (65). Along with CD45, they also express interleukin 7 receptor-α (IL-7Rα), neuropilin and CXCR5. In addition, comparison with mouse LTi cells, that are CD4+, human LTi cells are CD4− (65, 66). To test whether these molecules are expressed by EIV-E12 immunoreactive cells, double immunofluorescence was performed using chicken specific CD4, neuropilin, and IL-7Rα antibodies (58, 67, 68). Although CD4 and IL-7Rα are expressed widely on chicken T cells, and anti-neuropilin stained the blood vessels of the BF, cell surface molecules described for mouse LTi cells were not expressed by EIV-E12 cells in E12 BF (data not shown).

**Figure 7:**
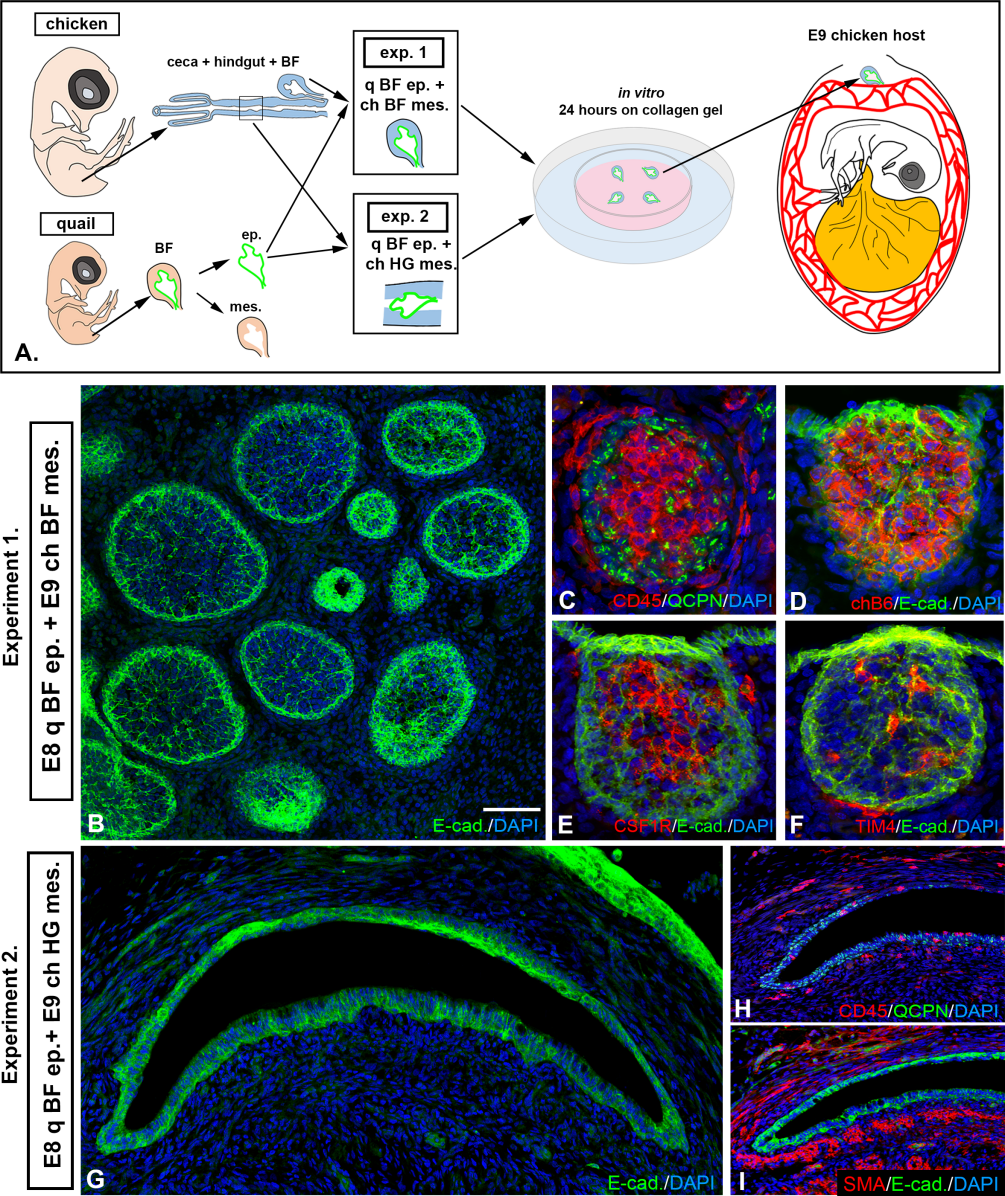
EIV-E12+ cells induce follicle bud formation. **(A)** To assess the follicle bud inducing capability of EIV-E12+ cells, quail-chick epithelial-mesenchymal recombination experiments were performed by recombining E8 quail BF epithelium with mesenchyme of bursa or hindgut origin (n=5). Recombinants were transplanted to E9 chick CAM for 9 days. **(B)** In Experiment 1 (n=5), where quail BF epithelium was recombined with chicken BF mesenchyme, normal follicle development was observed. **(C)** CD45+ hematopoietic cells colonize the developing follicles. **(C)** QCPN immunostaining confirms that the epithelium is quail derived. **(D-F)** Each follicle contains chB6+ B cells **(D)**, CSF1R+ dendritic cells **(E)** and TIM4+ macrophages **(F)**. **(G-I)** In Experiment 2 (n=5) where quail hindgut epithelium was recombined with bursa mesenchyme, no lymphoid follicles formed. **(G)** E-cadherin immunostaining reveals the differentiation of the epithelium. **(H)** QCPN immunostaining confirms that the epithelium is quail derived. CD45+ cells colonize the grafts, but clustering of the cells and follicle formation is not observed. **(I)** Staining for alpha-smooth muscle actin (SMA) showed muscularis layer located immediately under the epithelium. q, quail; ch, chicken; ep, epithelium; mes, mesenchyme; BF, bursa of Fabricius; HG, hindgut. Scale bar: 180 µm **(B-F)**, 350 µm **(G-I)**.

To study the inducer role of EIV-E12+ cells, quail-chicken epithelial mesenchymal recombination experiments were performed. The epithelium of the BF from E8 quail embryos was enzymatically separated from the underlying mesenchyme and recombined with E9 chick BF or hindgut mesenchyme. Chimeric recombinants were cultured overnight in 3D collagen gel, then transplanted onto the CAM of host E8 chicken embryos for 9 days (**Figure 7A**). Distinction of quail derived epithelium, from chicken derived mesenchyme was performed using the quail nucleus specific antibody, the QCPN (11), which is a valuable molecular tool for studying cell migration, fate map the stem cells in avian embryonic chimeras. As shown in **Figures 7B-F**, recombination of quail BF epithelium with chicken BF mesenchyme resulted in normal follicle development. CD45+ hematopoietic cells fill the quail derived QCPN+/E-cadherin+ epithelial follicles (**Figure 7C**) and each follicle is colonized by chB6+ B cells (**Figure 7D**), CSF1R+ dendritic cells (**Figure 7E**) and TIM4+ macrophages (**Figure 7F**). In recombinants using hindgut mesenchyme, the CAM grafts were able to receive host derived CD45+ cells (**Figure 1H**), but epithelial bud formation was not observed (**Figures 7G, I**). These results indicate that in the absence of EIV-E12+ cells epithelial-hindgut mesenchymal recombinants cannot differentiate into lympho-epithelial tissue.

## Discussion

The avian embryo is easily accessible for experimental manipulation and represents an exceptional vertebrate model organism for monitoring hemopoietic stem cell differentiation during lymphoid organogenesis. Although the chicken embryo has been widely used to study early hematopoiesis and lymphocyte differentiation, the ontogeny of lymphomyeloid organ forming cells in birds has not been specifically investigated. Similar to mammals, cellular components of the adaptive branch of the avian immune system are generated in distinct lympho-epithelial tissue microenvironments: the thymus harbors T cell development, while generation of immunocompetent B cells and the antibody repertoire occurs in an evolutionarily unique, gut-associated lymphoid organ, the bursa of Fabricius. The specialized microenvironment that guides maturation of B cell precursors in the bursa is dependent on incoming blood-borne cells that, after colonizing the bursal mesenchyme, migrate toward the luminal epithelium and induce follicle bud formation. Previously, the hematopoietic colonization of the BF was described as a two-step process: colonization of CSF1R+/MHCII+/74.3+ dendritic cell precursors to the epithelium drives the formation of epithelial follicle buds, followed by the homing of chB6+/CXCR4+ B cell precursors and the transition to lympho-epithelial tissue, where further maturation and proliferation of early B cells takes place (11, 25, 26, 28, 32, 48, 52). Despite extensive characterization of early bursal hematopoietic colonization, several questions arise regarding the identity of the first immigrating blood-borne population. In this study, we show that EIV-E12+ cells are among the earliest hematopoietic cells to colonize the bursa of the chicken embryo. They do not express lymphocyte, dendritic cell or macrophage lineage markers, suggesting a unique role in the development of lymphoid tissue architecture. Furthermore, our results support the view that EIV-E12+ cells act as an inducer cell of lymphoid follicle formation in the embryonic chicken BF.

After comprehensive immunocytochemical analysis of the developing chicken BF lymphoid follicles, we have identified a distinct population of CD45+/EIV-E12+ cells that appears in the bursal mesenchyme at E10 and colonizes the surface epithelium to induce follicle bud formation. According to previous histological studies, the first cells to enter the BF represent the CD45+/chB6- myeloid cells (macrophage and dendritic precursors) and, 48 hours later, the major influx of CD45+/chB6+ B cell precursors occurs (29, 48). Experiments using chick-quail chimeras and parabiosis proved that both types of precursor cells migrate into the BF primordium through the blood (26, 28). Pharr et al. (36) showed that an EIV-E12 immunoreactive cell population precedes the immigration of chB6 cells to the bursa epithelium and suggested that dendritic cells differentiate from the EIV-E12 cells. This cell surface marker however is not suitable to follow the fate of differentiating dendritic cells, because in post-hatch chicken the EIV-E12 specific antibody labels most of the bursal cells and is highly expressed in B cell-rich areas of avian lymphoid organs (36, 62). Performing extensive double immunostainings with chicken lympho-myeloid specific antibodies and EIV-E12 immunoelectron microscopy we have confirmed that EIV-E12+ cells colonize the bursal epithelium first, and co-express CD45, but do not share dendritic cell, lymphocyte, or macrophage markers. In this study, we also used transgenic *CSF1R-*eGFP embryos to permanently label and fate map the migration and differentiation of the bursa specific dendritic cells (BSDC). Characterization of *CSF1R-*eGFP transgenic chicken embryos confirmed the presence of the EIV-E12+*/CSF1R-*eGFP- bursal cells that precede the entry of CD45+/*CSF1R-*eGFP+ dendritic cell precursors to the follicle buds. Once EIV-E12+*/CSF1R-*eGFP- cells reach the epithelium, sequential formation of follicle buds takes place, followed by the entry of *CSF1R-*eGFP expressing cells that together shape the follicular microenvironment prior to colonization of chB6+/IgM+ B cell precursors. Using duck-chicken CAM grafting experiments, we support the observation that EIV-E12+ cells present in the early chicken BF represent a transient cell population in the epithelial follicles, without the capacity to differentiate to either dendritic cells, macrophages, or B cells. These results strongly support the idea that, similar to mammalian lymphoid tissue inducer cells (LTi), the EIV-E12+ cell is the first to colonize the embryonic BF and is the key element for inducing and orchestrating lymphoid follicle formation.

Cyclophosphamide-induced chemical B cell depletion and CRISPR/Cas9 gene-edited RAG1 knockout systems, have been extensively used to generate immunodeficient chicken model systems (42, 69–72). Similarly, systemic or local testosterone-propionate treatment results in the development of birds lacking a bursa by preventing the differentiation of bursal secretory dendritic cells and consequently the formation of dendro-epithelial tissue that is indispensable for further maturation of B cells within the bursa (27, 64). Despite the absence of these blood-borne populations in both systems, formation of abortive bursal follicle buds was reported with scattered dark cells present inside the rudimentary follicles (27, 73). Similarly, testosterone-propionate mediated depletion of 74.3+ dendritic cells in the embryonic bursa does not influence clustering of EIV-E12+ cells and has no inhibitory effect on early stages of follicle bud formation (64). In this study we confirm that treatment of embryonic bursa with AMD3100, the selective antagonist of the CXCR4 chemokine receptor, results in the formation of B cell depleted bursa follicles (31), but does not affect immigration of EIV-E12+ cells and CSF1R+/74.3+ dendritic cells into the developing follicles. We therefore conclude that clustering of EIV-E12+ cells in the epithelium represents the earliest step of folliculogenesis in the chicken bursa of Fabricius, and does not require involvement of any other lympho-myeloid cell types. Quail-chick epithelial mesenchymal recombination experiments further support the inducer role of EIV-E12+ cells, with the capacity of these cells to colonize epithelium derived tissue compartments and induce the formation of integral follicles.

**Figure 8:**
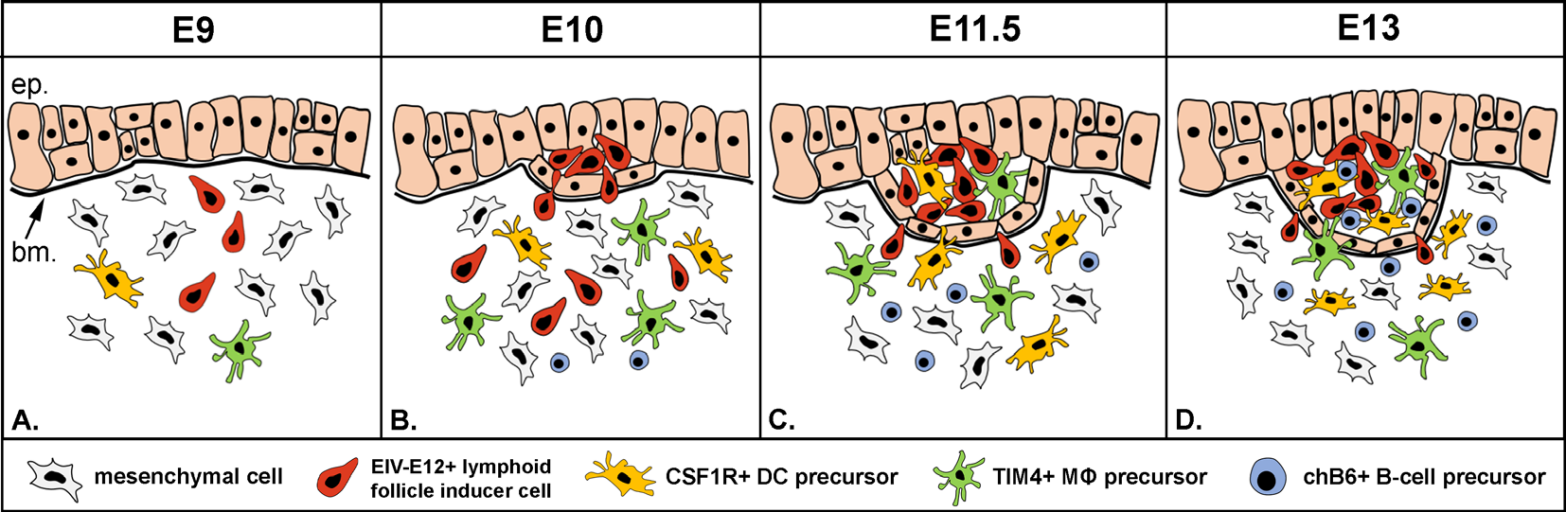
New model of folliculogenesis in the bursa of Fabricius. **(A, B)** Step 1: Blood-borne CD45+/EIV-E12+ lymphoid follicle inducer cells are the first to reach the surface epithelium on E10, cross the basement membrane and induce lymphoid follicle bud formation. **(C)** Step 2: CSF1R+ dendritic cells and TIM4+ macrophage precursors colonize the developing follicle buds on E11.5. **(D)** Step 3: chB6+/IgM+ B cell precursors first enter the follicle buds on E13. ep, epithelium; bm, basement membrane; DC, dendritic cell; MΦ, macrophage.

Based on the findings shown in this study, we propose a novel three-step colonization model for BF development (**Figure 8**). Step 1: the mesenchymal cells condensed under the bursa surface epithelium are primed by a yet unknown signal to secrete CXCL12, alkaline phosphatase and proteases, able to receive CD45+/EIV-E12+ cells (31, 74, 75). Interaction of EIVE12+ cells with surface epithelium marks the sites for follicle bud formation. Step 2: CD45+/CSF1R+/74.3+ cells colonize the follicle buds, create the transient dendro-epithelial tissue and later give rise to the mature bursal dendritic cells. Dendritic cell precursors accumulate in the follicle buds at the same time with CD45+/TIM4+ macrophages. Step 3: B cell precursors enter into the growing follicles. This multistep sequential model is similar to mammalian lymphoid organogenesis, which is also a complex developmental process characterized by sequential steps, including: 1) initial interaction between mesenchymal derived lymphoid tissue organizer (LTo) and blood-borne CD45+ lymphoid tissue inducer cells, 2) followed by differentiation of antigen presenting follicular dendritic cells, and 3) migration of mature lymphocytes into the B- and T-cell dependent compartments. These steps together lead to the development of Peyer’s patches and lymph nodes, classical secondary lymphoid organs (76). Similar to mesenchymal LTo cells described in mammals, the earliest phases of BF development consists of migration and condensation of pale mesenchymal cells underneath the surface epithelium (27), which correlates with the appearance of alkaline phosphatase and protease activity within the subepithelial mesenchymal compartment. These mesenchymal cells localized in the subepithelial mesenchyme are thought to represent the precursor for mesenchymal reticular cells of adult lymphoid follicles (27), and we hypothesize that it may also represent the LTo cell population for lymphoid follicle bud initiation.

Embryonic and postnatal LTi cells that drive development of the secondary lymphoid organs and orchestrate tertiary lymphoid structure formation in mammals are a highly heterogeneous population, with phenotypic markers that vary among species (77). Neuropilin, CXCR5 and IL-7Rα expression has been described on mammalian LTi cells, but their expression in chicken embryos is not known. Chicken-specific antibodies do not cross-react with EIV-E12 cells, suggesting that the immunophenotype of avian LTi cells may differ from that of mammals. Major signals involved in the function and migration and of mammalian LTi cells to sites of secondary lymphoid organ formation include RANK-RANKL (78, 79), lymphotoxin-LTβR (80) and CXCR5-CXCL13 mediated processes (81). Expression of these molecules has not yet been described in developing lymphoid organs in birds. Indeed, the chicken genome lacks the genes for lymphotoxins and their receptors (82). Mammalian LTi cells are key producers of bioactive lymphotoxin α1β2 (83), and mice deficient for lymphotoxin or its receptors lack Peyer’s patches, lymph nodes and tertiary lymphoid tissues (83, 84). Therefore, these data suggest that functionally avian LTi cells differ substantially from their mammalian counterpart, and uncovering the developmental cues that guide migration and potentiate function of LTi cells in the avian model system will require further studies. CXCL13 or CCL20, and their cognate receptors CXCR5 and CCR6, facilitate inductive steps of secondary lymphoid organ formation in mammals (81, 85) and are also present in chicken (86). Single cell transcriptomic analyses revealed several cell surface molecules (87), among which CXCR5, the chemokine receptor for CXCL13 was uniformly expressed by LTi cells and played a predominant role in directing them to developing mouse lymphoid structures (81, 85). CXCR5/CXCL13 expression was also reported to be present on CD4+ cells isolated from the adult bursa (88), but not in the embryo. Taking advantage of transcriptomic platforms, complemented with cell sorting and adoptive cell transfer experiments, will stimulate future studies of the developmental mechanisms that guide avian LTi cell differentiation during lymphoid organogenesis, which seems to be an evolutionarily conserved multistep process among higher vertebrates.

## Data Availability

The raw data supporting the conclusions of this article will be made available by the authors, without undue reservation.
