# Supplementary material for: Characterization and ontogeny of a novel lymphoid follicle inducer cell during development of the bursa of Fabricius
Source: Front Immunol. 2024 Oct 21;15:1449117. doi: 10.3389/fimmu.2024.1449117 (PMC11532080; doi:10.3389/fimmu.2024.1449117)
Supplement: Supplementary file 1 [file DataSheet1.pdf]

Supplemental figure 1.

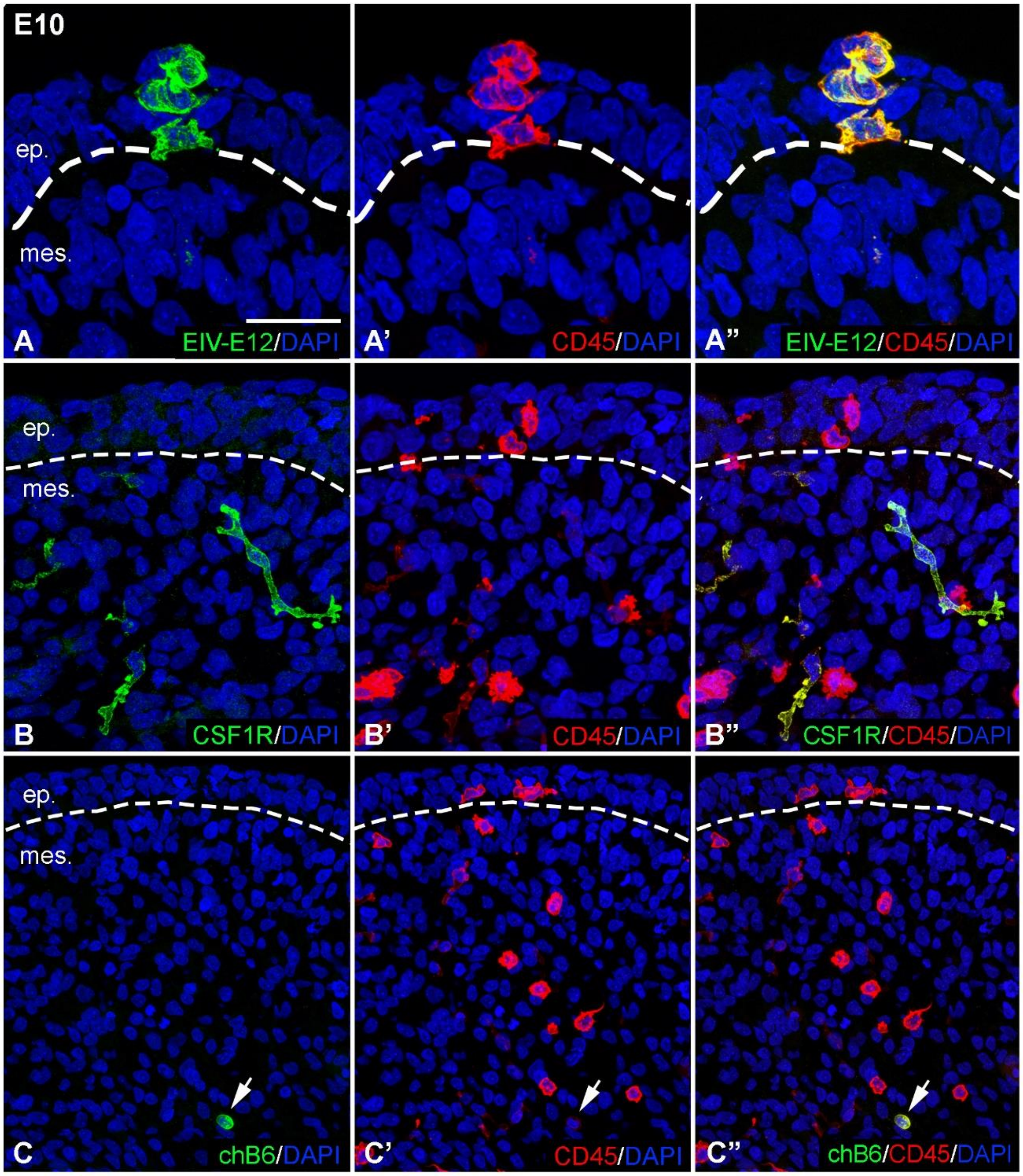


**Fig. S1. Hematopoietic cells colonizing the bursa epithelium.** Consecutive sections from bursa of Fabricius of E10 embryo immunostained with CD45 mAb shows numerous hematopoietic cells distributed throughout the mesenchyme. Inside the epithelium (dashed line), large groups of round CD45+/EIV-E12+ cells form clusters, which do not express CSF1R (B-B’) and chB6 (C-C”) antigens. Scale bar: 20 µm (A-A”), 30 µm (B-B”), 40 µm (C-C”).

Supplemental figure 2.

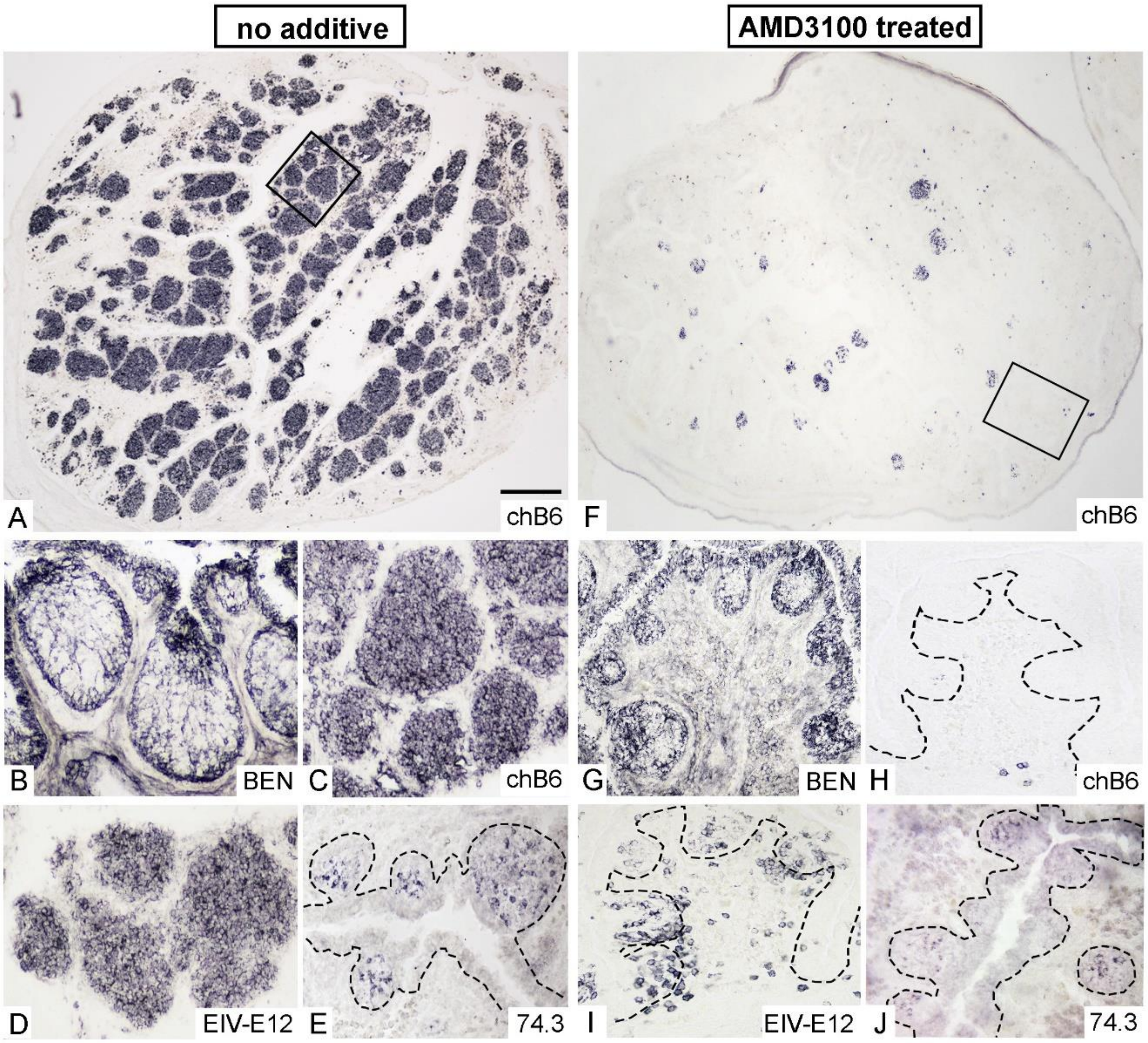


**Fig. S2. Depletion of B cells does not prevent follicle bud formation**. E9 bursa of Fabricius was injected with AMD3100 (n=9) or vehicle (no additive, n=6) and cultured on the chorioallantoic membrane (CAM) of E9 host chick embryo. (A) Non-treated bursa of Fabricius shows a well-developed lymphoid follicular structure. (B, C) BEN+ epithelial follicles are filled with chB6+ B cells. (D) Distribution of EIV-E12+ cells correspond to B-dependent follicles. (E) 74.3+ dendritic cells are observed in the follicles. (F) Addition of AMD3100 inhibits B cell colonization of the bursa of Fabricius. Boxed area in F is shown in H. (G) BEN immunoreactivity labels the bursal epithelium with growing epithelial buds towards the mesenchyme. (I) EIV-E12+ cells are present in large numbers in the epithelial follicle buds. (J) AMD3100 treatment did not influence the colonization of 74.3+ dendritic cells to the bursa follicles. Scale bar: 280 µm (A), 80 µm (B-E), 220 µm (F), 50 µm (G-J).

Supplemental figure 3.

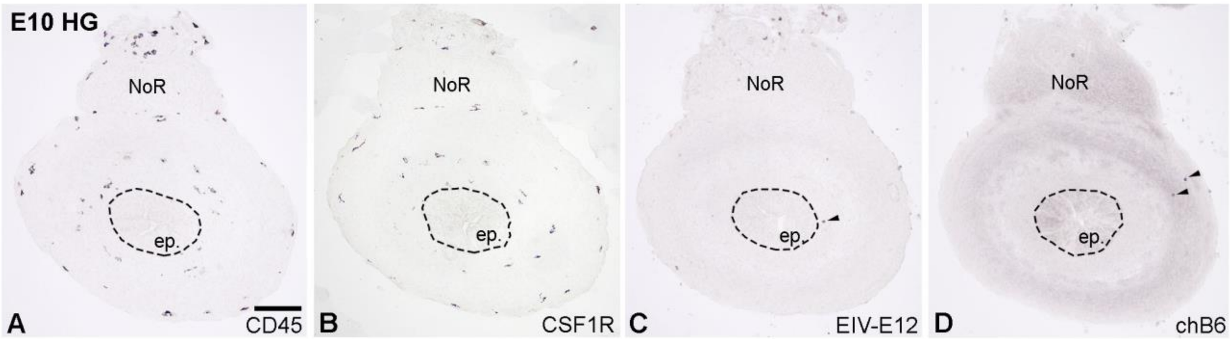


**Fig. S3. Distribution of hematopoietic cells in the E10 hindgut.** (A) CD45+ cells with a ramified morphology are present in the subepithelial and peripheral mesenchymal compartments. (B) Consecutive sections stained with CSF1R (B), EIV-E12 (C), and chB6 (D) antibodies reveal the presence of few immunoreactive cells (C,D arrowheads) scattered in the HG mesenchyme. ep, epithelium; NoR, nerve of Remak. Scale bar: 110 µm.
